# The effectiveness of preoperative rehabilitation programmes on postoperative outcomes following anterior cruciate ligament (ACL) reconstruction: a systematic review

**DOI:** 10.1186/s12891-020-03676-6

**Published:** 2020-10-03

**Authors:** Hayley M. Carter, Chris Littlewood, Kate E. Webster, Benjamin E. Smith

**Affiliations:** 1 Department of Physiotherapy, London Road Community Hospital, University Hospitals of Derby and Burton NHS Foundation Trust, London Road Community Hospital, Derby, DE1 2QY UK; 2 Department of Health Professions, Faculty of Health, Psychology and Social Care, Manchester Metropolitan University, Manchester, UK; 3 School of Allied Health, Human Services and Sport, La Trobe University, Melbourne, VIC 3086 Australia; 4 Division of Rehabilitation, Ageing and Wellbeing, School of Medicine, University of Nottingham, Nottingham, UK

**Keywords:** Anterior cruciate ligament (ACL), Rehabilitation, Postoperative outcomes, Systematic review

## Abstract

**Background:**

To explore the effectiveness of preoperative rehabilitation programmes (PreHab) on postoperative physical and psychological outcomes following anterior cruciate ligament reconstruction (ACLR).

**Method:**

A systematic search was conducted from inception to November 2019. Randomised controlled trials (RCTs) published in English were included. Risk of bias was assessed using Version 2 of the Cochrane risk-of-bias tool, and the Grading of Recommendations Assessment system was used to evaluate the quality of evidence.

**Results:**

The search identified 739 potentially eligible studies, three met the inclusion criteria. All included RCTs scored ‘high’ risk of bias.

PreHab in all three RCTs was an exercise programme, each varied in content (strength, control, balance and perturbation training), frequency (10 to 24 sessions) and length (3.1- to 6-weeks).

Statistically significant differences (*p* < 0.05) were reported for quadriceps strength (one RCT) and single leg hop scores (two RCTs) in favour of PreHab three months after ACLR, compared to no PreHab. One RCT reported no statistically significant between-group difference for pain and function. No RCT evaluated post-operative psychological outcomes.

**Conclusion:**

Very low quality evidence suggests that PreHab that includes muscular strength, balance and perturbation training offers a small benefit to quadriceps strength and single leg hop scores three months after ACLR compared with no PreHab. There is no consensus on the optimum PreHab programme content, frequency and length. Further research is needed to develop PreHab programmes that consider psychosocial factors and the measurement of relevant post-operative outcomes such as psychological readiness and return to sport.

**Trial registration:**

PROSPERO trial registration number. CRD42020162754.

## Background

The anterior cruciate ligament (ACL) is the most commonly injured ligament in the knee with annual incidence rates of ruptures reported at 68.6 per 100,000 person-years [1]. ACL ruptures are commonly treated with surgical reconstruction [2] which aims to restore knee stability and maximise functional capacity to allow individuals to return to their preinjury level of physical activity [3]. Prior to ACLR, preoperative rehabilitation, commonly termed prehabilitation (PreHab), has been suggested to physically and mentally prepare patients for surgery and postoperative rehabilitation [4, 5].

No previous systematic review has specifically evaluated the effectiveness of PreHab on postoperative outcomes. A 2017 systematic review did investigate what the authors termed ‘pre-operative rehabilitation’, but of the included eight RCTs only two included post-operative outcomes and not all RCTs included surgery in the treatment pathway [6]. This review concluded that rehabilitation following ACL injury is effective for improving function, strength and hamstring reflex latency but these results are not exclusive to patients following surgery. Further, the review did not include the effects of pre-operative rehabilitation on return to pre-injury levels of sport; the ultimate goal for most patients following surgery [7].

Return to sports participation after ACLR is commonly cited in the literature to be inadequate despite patients achieving a successful functional outcome [3, 8, 9]. A recent cohort study revealed that at 1-year post-surgery, only 24% of individuals (*n* = 675) had returned to their pre-injury level of sport despite 91% reporting preoperatively to expect to return [10]. Reasons for failing to return have also been reported in the literature, with psychological barriers commonly cited as potential causes [3, 11–17]. A number of authors have identified the need to address these psychological barriers, however, research to date has focused on using psychological factors as predictors of return to physical activity outcomes, rather than considering them in complex intervention development work [3, 10, 11, 16–19]. Return to sport following ACL rupture remains a complex clinical problem with no current validated guidelines to inform a ‘safe return’ decision. In addition to considering patients’ psychological readiness for return, there are a plethora of physical tests that are combined to form a ‘test battery’ and it is well documented that the proportion of patients who pass these test batteries, is typically low [20].

Therefore, the primary aim of this systematic review was to evaluate the effectiveness of PreHab on physical and psychological outcomes following ACLR.

## Methods

This systematic review was registered with the International Prospective Register of Systematic Reviews (PROSPERO 2020 CRD42020162754; https://www.crd.york.ac.uk/prospero/display_record.php?ID=CRD42020162754) and reported following the PRISMA statement (available in supplementary file 1) [21].

### Search strategy

Articles were identified via an electronic search of the following six databases: CINAHL, AMED, PsycINFO, Medline and SPORTDiscus via EBSCOhost and Web of Science from inception to November 2019. Databases were searched in addition to the reference lists of included articles and the grey literature via OpenGrey, ClinicalTrials.gov and WHO International Clinical Trials Registry Platform. The search strategy used a range of keywords in three categories (1) ACL (2) preoperative interventions (3) post-operative outcomes and were combined using Boolean operators (search strategy available in supplementary file 2).

### Study selection

Articles were imported into a reference management software and duplicates were removed. One reviewer (HC) independently reviewed titles and abstracts for eligibility against predetermined criteria. The full text articles were independently screened by two reviewers (HC and BS). Inclusion agreement was 100%. For inclusion, the studies had to meet the eligibility criteria shown in Table 1.

**Table 1:** Eligibility Criteria

Participants	Any age or sex undergoing primary ACLR
Intervention	Any therapy intervention completed prior to ACLR
Outcomes	Reported post ACLR:
	**Physical**
	Any outcome related to pain, disability or function, including but not exclusive to: joint range of movement, muscular strength, single leg hop distance and return to sport/physical activity
	**Psychological**
	Any outcome related to psychological status or well-being such as anxiety or depression scores
Study Design	Randomised controlled trials (RCTs) only
Language	English only

### Assessment of methodological quality

The methodological quality of each study was independently assessed by 2 reviewers (HC and BS) using version 2 of the Cochrane risk-of-bias (ROB-2) tool for RCTs, with a third reviewer available to resolve discrepancies.

The Cochrane Risk of Bias tool was originally developed in 2008, and most recently updated in 2019 to the ROB-2 tool [22]. It includes five domains that aim to assess bias relating to: the randomisation process, deviations from intended intervention, missing outcome data, measurement of the outcome and selection of the reported result. The excel implementation tool was used which allows an answer of ‘yes’, ‘probably yes’, ‘probably no’, ‘no’ or ‘no information’ to be inputted for each question with an algorithm used to suggest the level of bias for each domain. An overall judgement from the five domains is calculated to determine ‘low risk’, ‘some concerns’ or ‘high risk’ for each individual study. Percentage agreement between the two reviewers (HC and BS) for the individual risk of bias domains for the Cochrane ROB-2 tool was 83%; calculating Cohen’s kappa statistic, the agreement was k = 0.73, which is considered substantial [23]. Disagreements were resolved through discussion.

### GRADE assessment

The Grading of Recommendations Assessment, Development and Evaluation (GRADE) system was also used to rate each outcome [24, 25]. The outcomes were assessed and reported as one of four grades, shown in Table 2.

**Table 2:** GRADE Quality of Evidence

Grade	Definition
High	We are very confident that the true effect lies close to that of the estimate of the effect.
Moderate	We are moderately confident in the effect estimate: The true effect is likely to be close to the estimate of the effect, but there is a possibility that it is substantially different
Low	Our confidence in the effect estimate is limited: The true effect may be substantially different from the estimate of the effect.
Very Low	We have very little confidence in the effect estimate: The true effect is likely to be substantially different from the estimate of effect

With reference to the GRADE handbook [25], each outcome starts at a ‘high’ grade as all derive from a RCT study design. Outcomes are then downgraded as appropriate based on five domains (1) Study limitations (2) Inconsistency (3) Indirectness (4) Imprecision and (5) publication bias. Two reviewers (HC and BS) assessed these domains and agreement was reached by consensus. Further detail regarding the judgments made are included in supplementary file 3. Publication bias was not assessed as funnel plot asymmetry is recommended to only be used when there are ten or more studies included [26].

### Data extraction

Data were extracted by one reviewer (HC) in relation to study location, sample size and population, intervention and setting, outcome measures and data collection time points, and results. Where necessary, authors were contacted to request further data where that reported in the published article or supplementary material were deemed insufficient. The data extraction table was verified by a second reviewer (BS).

### Data analysis

The extracted data were assessed for clinical heterogeneity. Due to the differences in exercise interventions investigated, study populations and outcome measures, it was deemed that included studies were not homogenous, and thus, a meta-analysis could not be completed. Quadriceps strength was assessed in all three RCTs but the assessment method varied between studies. Single leg hop for distance was assessed in two RCTs with a standardised test; therefore, both authors of these RCTs were contacted via the corresponding email provided on publication for raw data [27, 28]. One author [27] responded and provided the original data for the study however, the other author [28] did not respond after three months. Where statistical significance was found, the standardised mean difference (SMD) was calculated to determine effect size using the OpenMetaAnalyst software [29]. As per Cohen [30], the effect size interpretation was greater than or equal to 0.2 for ‘small’, greater than or equal to 0.5 for ‘medium’ and greater than or equal to 0.8 for ‘large’.

## Results

**Fig. 1:**
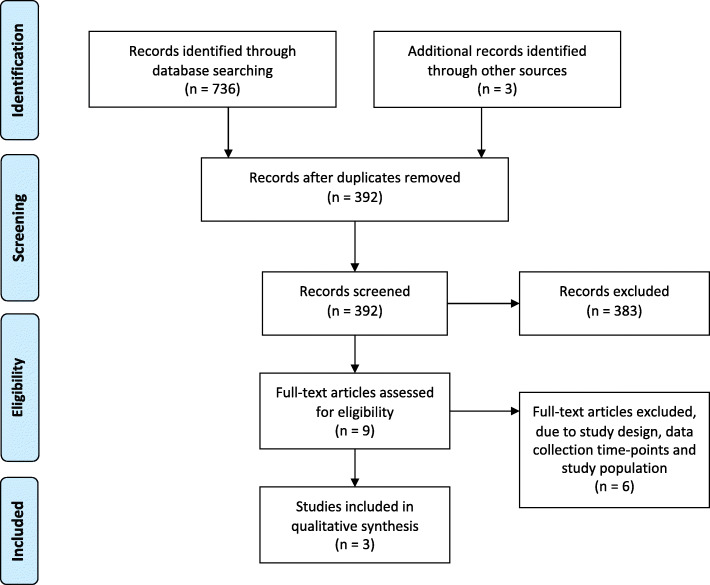
PRISMA 2009 Flow Diagram

The study selection process is presented in Fig. 1. The initial database search yielded 736 articles. After duplicates were removed, 392 articles were screened for inclusion. No additional articles were found from the screening of unpublished searches. After title and abstract screening, eight full-text articles were assessed for eligibility. Six were excluded due to study design (not RCT), data collection time-points (not all studies assessed participants post operatively) and study population (not all participants underwent ACLR) (supplementary file 4). Three further articles were found from reference list screening of which two were duplicates; the remaining article was deemed to meet the inclusion criteria by both reviewers (HC and BS) and was included. The total number of RCTs included was three [27, 28, 31].

### Characteristics of the included studies

The characteristics of the three included RCTs are summarised in Table 3.

**Table 3:** Characteristics of Included Studies

Authors, Year of Publication and Study Location	Sample Size and Study Population	Intervention and Setting	Outcome Measures and Data Collection Time Points	Results
Hartigan, Axe and Snyder-Mackler (2009) [31] *USA*	*n* = 19 13 males, 6 females. Age range 17–50. Subjects were recruited from the University of Delaware Physical Therapy Clinic, USA, and were referred into the study by one surgeon. Inclusion criteria: (a) Regular participation in Level I and II activities (b) Subject classified as ‘non-copers’ following a screening examination Exclusion criteria: (a) Full thickness chondral defect > 1 cm (b) repairable meniscal tears (c) Concomitant grade III ruptures to other knee ligaments	Subjects were randomly assigned to 2 groups: **1. Perturbation group** (PERT) (n = 9) 6 males and 3 females (28 ± 10.7 years), averaging 9.8 ± 9.5 weeks from the time of injury to the screen **2. Strengthening group** (STR) (*n* = 10) 7 males and 3 females (30 ± 9.4 year), averaging 12.6 ± 13.1 weeks from the time of injury to the screen No subjects exercised their lower extremities outside of therapy while participating in the preoperative intervention phase. **1. PERT** group received 10 sessions of physical therapy including specialized neuromuscular exercises involving systematic translation of support surfaces and progressive quadriceps strength training (average 3.7 weeks to complete). The University of Delaware guidelines for perturbation training were followed. **STR** group received 10 sessions of progressive quadriceps strength training only (average 3.1 weeks to complete). After the 10 preoperative sessions, ACLR was performed using either semitendinosus-gracilis autograft or soft tissue allograft. The University of Delaware postoperative ACL protocol was followed regardless of group.	**Quadriceps strength index** (involved/uninvolved side) was calculated and reported as a percentage using the highest quadriceps maximum volitional isometric contraction (MVIC) force output from each limb. **Knee excursion** (obtained by calculating peak knee extension minus peak knee flexion) during the mid-stance phase of gait were measured. Data were collected **prior to the intervention** and at **6 months postoperatively**.	**Quadriceps Strength** Quadriceps strength indexes improved over time *(F = 16.5, observed power = 0.961, p = 0.002).* Quadriceps strength indexes before intervention *(Pert: 87.2%; Str: 75.8%)* improved 6 months after ACL reconstruction in both groups *(Pert: 97.1%; Str: 94.4%).* *No between group differences were reported.* **Knee Excursion** Significant differences were found in knee excursions between limbs *(F = 15.98, observed power = 0.96, p = 0.001)* and over time (*F = 7.52, observed power = 0.73, p = 0.014).* Knee excursions at mid-stance were smaller on the involved side prior to surgery in both groups The involved limb moved through less flexion in the perturbation group *(Mean: 5.98; 95% CI: 10.2 to 1.5; p = 0.026)* and strength group *(Mean: 5.68; 95% CI: 10.5 to 0.06; p = 0.031).* The perturbation group demonstrated an increase in knee excursion at midstance compared to the uninvolved side, resulting in no significant difference between limbs 6 months after surgery *(Mean: 3.58; 95% CI: 8.3 to − 1.4; p = 0.14).* The mid-stance knee excursions continued to be significantly different between limbs in the strength group 6 months after surgery *(Mean 7.08; 95% CI: 11.6 to 2.5; p = 0.007).* *No between group differences were reported.*
Kim, Hwang and Park (2015) [28] *Korea*	*n* = 80 80 males, 0 females. Mean age 27.8 ± 5.7 Subjects were recruited from the Samsung Medical Orthopaedics Centre, Sungkyunkwan, South Korea. Inclusion criteria: (a) Male (b) Aged 20–35 (c) isolated ACL rupture Exclusion criteria: (a) Previous ACLR or meniscus repair (b) Injury to other ligaments in the same knee (c) Associated fractures	Subjects were randomly assigned to 2 groups: **1. Preoperative exercise group** (PEG) (*n* = 40) 2. **No preoperative exercise group** (NPEG) (n = 40) **PEG** participated in a 4-week exercise programme preoperatively and in a 12-week postoperative programme. The preoperative programme focused mainly on strengthening with particular attention paid to the quadriceps muscle, functional balance, muscle control and co-contraction. The exercise programme was however, adapted to meet patient specific conditions and needs, but included stationary bike, range of movement exercises, open and close chain strengthening exercises and balance/proprioception exercises. **NPEG** participated in the 12-week postoperative programme only. Postoperatively: • 0–2 weeks: operated limb immobilised in a functional brace, subjects instructed to complete straight leg raises and quadriceps setting exercises. • 2–4 weeks: subjects were allowed to complete partially weight bearing exercises and move through full knee joint range of movement • 4+ weeks: subjects able to complete closed chain exercises	**Knee extensor strength deficit** (calculated as the percentage difference between the uninjured and injured limb) and the **limb symmetry index (LSI)** for **single leg hop distance** were measured at **4 weeks before surgery** and **3 months after surgery**. Knee extensor strength was measured through the range of 0-90^o^ at an angular speed of 60^o^/s, 4 repetitions completed at an angular speed of 180^o^/s, with 20 repetitions completed to calculate average power. The highest peak torque value for each velocity was compared with the uninjured side and described as percent of strength deficit. The mean average distance was calculated for the single leg hop test and was quantified by LSI using the formula: distance for uninjured leg/distance for injured leg) × 100.	**Knee extensor deficit (%) 60**^**o**^**/s**: Preoperative: 22.8 ± 13.7 for PEG and 23.5 ± 15.8 for NPEG. Postoperative: 28.5 ± 9.0 for PEG and 36.5 ± 10.7 for NPEG **Knee extensor deficit (%)180**^**o**^**/s**: Preoperative: 16.6 ± 10.6 PEG and 17.5 ± 11.9 NPEG Postoperative: 23.3 ± 9.0 PEG and 27.9 ± 12.6 NPEG Knee extensor strength deficits were significantly different between the groups at both angular velocities *(60*^*o*^*/s; p = 0.018, 180*^*o*^*/s; p = 0.033)*. Subjects in the PEG showed a significantly greater improvement in postoperative strength than the NPEG at 60^o^/s and 180^o^/s. **Single leg hop LSI (%)**: Preoperative: 75.1 ± 10.3 PEG and 76.5 ± 8.9 NPEG Postoperative: 85.3 ± 7.4 PEG and 80.5 ± 9.6 NPEG The PEG showed significant improvement in the single leg hop distance test (*p* = 0.029) compared to NPEG.
Shaarani et al., (2013) [27] *Ireland*	*n* = 23 (3 drop-outs) Mean age:Exercise group 27.55 ± 7.85 Control group 32 ± 8.3 Subjects were recruited from 2 orthopaedic centres, Dublin, Republic of Ireland. Inclusion criteria: (a) Male (b) Aged 18–45 (c) Isolated ACL tear Exclusion criteria: (a) Associated fractures (b) Meniscal repair (c) Associated collateral ligament injury requiring repair/reconstruction (d) comorbidities that would be contraindicated with high physical exertion (e) living outside the Greater Dublin area	Subjects were randomly assigned to 2 groups: 1. 6-week gym- and home-based preoperative exercise (prehabilitation) group (*n* = 11) 2. Control group (*n* = 9) There was no significant different in age, height, weight, body mass index and Tegner activity level before and after injury between the groups at baseline. The **prehabilitation** group completed a 6-week supervised resistance and balance training programme. This consisted of 4 exercise periods per week: 2 supervised gym sessions and 2 supervised home sessions. The **control** group were not discouraged to do any exercise or normal activity of daily living but were asked to keep a record of exercise activity performed during the weeks before surgery. All patients had an ACLR performed by one surgeon using a standard bone-patellar tendon-bone graft. Both groups undertook a standard postoperative physiotherapy programme. This was split into 6 phases over a 12 week period and progressed from early exercises to improve knee joint range of movement, weight bearing ability and gait to increasing strength, proprioception and balance.	**Single leg hop distance** (the best distance from 3 jumps), **quadriceps** and **hamstring peak torque** (measured at an angular speed of 90^o^/s), and **quadriceps cross sectional area** (CSA) (measured using magnetic resonance imaging [MRI]), were assessed at **baseline**, **before the ACLR** and **12 weeks postoperatively**. Pain and function were also assessed using the **Modified Cincinnati Knee Rating System** at all 3 time points. The **Tegner** activity level was also completed although authors lacked clarity regarding time points taken. The **Tegner-Lysholm Knee Score** was also taken at all 3 time points.	**Single Leg Hop Distance** The mean preoperative score (mean ± SD) was higher for the prehabilitation group (183.1 ± 15.55) compared to the control group (156.0 ± 42.98) (*p* = 0.13). At 12-weeks postoperatively, the single leg hop scores were reduced for both groups but the prehabilitation group (144.91 ± 15.52) had significantly higher scores compared to the controls (113.33 ± 25.54) (*p* = 0.001). The prehabilitation group had a statistically significant improvement in single leg hop distance preoperatively compared to baseline (p = 0.01). **Quadriceps Peak Torque** Quadriceps peak torque increased significantly from baseline to the preoperative time point in the injured (p = 0.001) and uninjured limb (*p* = 0.009). In the prehabilitation group, there was a significant decrease in quadriceps peak torque of the injured limb at 12 weeks postoperatively compared with baseline (*p* = 0.042) and preoperative (*p* < 0.001) time points. There were no statistically significant differences between the prehabilitation and control group for the injured limb at any time point (mean [SD], pre-operation: 151.1 [30.21] and 138.7 [43.92], post-operation: 102.1 [22.18] and 89.27 [34.70] for exercise and control groups respectively). **Hamstring Peak Torque** Hamstring peak torque increased significantly in the injured limb from baseline to preoperatively for both the prehabilitation group (*p* = 0.034) and control group (p < 0.001). No significant differences were found for hamstring peak torque between groups at both pre- and post-operative time points. **Modified Cincinnati Knee Rating System** The prehabilitation group scores increased significantly from baseline (62.6) to the preoperative time point (76.5) (*p* = 0.004) to 12-weeks postoperatively (85.3) (p = 0.001). The mean score at 12 weeks postoperatively was significantly higher (p = 0.004) for the prehabilitation group (85.3) compared with the controls (77.7). **Tegner-Lysholm Knee Score** The prehabilitation group scores increased significantly at all time points from baselines (*p* = 0.006). There was no significant difference between group scores at any time point. **Time to Return to Sport (RTS)** The mean time (SD) to RTS was 42.5 weeks (10.46) for the control group and 34.18 weeks (4.14) for the prehabilitation group. This difference was not significant (*p* = 0.055).

The three RCTs investigated a total of 122 participants, of which 116 (95%) were male. Two RCTs excluded female participants [27, 28]. Two RCTs included a PreHab group compared to a control group who received no preoperative exercise programme [27, 28]. The remaining RCT compared two different preoperative exercise protocols [31].

All RCTs evaluated quadriceps strength. Two RCTs utilised a single leg hop for distance test [28, 31]. One RCT assessed knee excursion during the mid-stance phase of gait to report between limb symmetry [31]. One RCT also reported hamstring strength, Tegner-Lysholm Score, Modified Cincinnati Knee Rating System and time to return to sport (RTS) [27]. All RCTs included pre- and post-operative outcome measures although time-points at which they were assessed varied. No RCT utilised a psychological outcome measure.

All RCTs included pre- and post-operative outcome measures, although time-points varied between studies; Hartigan, Axe and Snyder-Mackler [31], pre-intervention and 6-months post-operatively; Kim, Hwang and Park [28] 4-weeks pre-operatively (pre-intervention) and 3-months post-operatively; Shaarani et al. [27] prior to the intervention, preoperatively (post intervention) and 3-months post-operatively.

### Assessment of methodological quality and GRADE

**Fig. 2:**
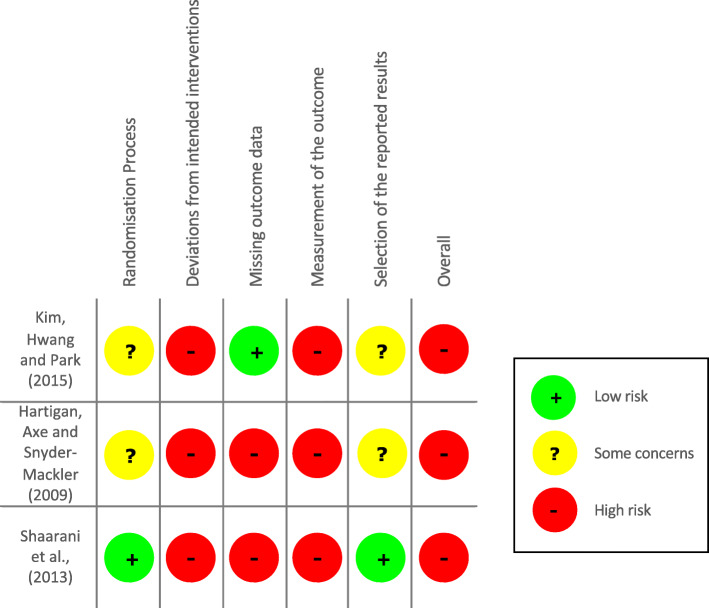
Risk of Bias Summary

**Fig. 3:**
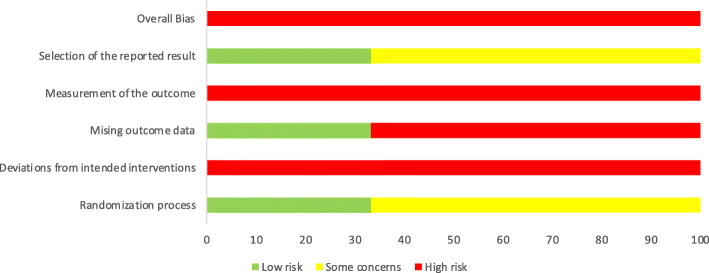
Risk of Bias Graph

A summary of the risk of bias assessment, using the Cochrane ROB-2 tool, is shown in Figs. 2 and 3.

Overall, all RCTs scored a high risk of bias and all RCTs had at least two ‘high risk of bias’ domain scores (Fig. 2). All studies were high risk for ‘deviations from intended interventions’ and ‘measurement of the outcome’ (Fig. 3). Common omissions across studies for these two domains included lack of detail with regard to study protocol and lack of blinding of participants and study personnel.

Shaarani et al. [27] scored low risk for the ‘randomisation process’ domain as they reported sufficient detail with regard to the randomisation of study participants whereas Kim, Hwang and Park [28] and Hartigan, Axe and Snyder-Mackler [31] did not. All three RCTs failed to report whether deviations arose from the intended interventions and only the protocol for Shaarani et al. [27] study was available for comparison between final study procedures and that planned in the protocol.

Only one RCT [28] scored ‘low risk’ for ‘missing outcome data’ as all participants could be accounted for in the data table provided. Hartigan, Axe and Snyder-Mackler [31] failed to declare a drop-out rate and although Shaarani et al. [27] reported 3 drop-outs (13% of study participants), they excluded their data from analysis and thus did not utilise intention-to-treat approach, introducing a high risk of bias.

The results of the GRADE assessment are shown in Table 4. The quality of evidence was rated as ‘very low’ for all outcomes due to trial design limitations, heterogeneity and low participant numbers for all outcomes.

**Table 4:** GRADE Summary of Findings Table

Summary of Results		GRADE Assessment				
Outcome	Number of Participants (studies)	Study Design	Inconsistency	Indirectness	Imprecision	Quality
Quadriceps Strength	122 (3)	Limitations^a^	Inconsistency^c^	Indirectness^e^	Imprecision^f^	⨁◯◯◯ Very Low
Single Leg Hop Distance	103 (2)	Limitations^a^	Inconsistency^c^	Indirectness^e^	Imprecision^f^	⨁◯◯◯ Very Low
Gait Asymmetry	19 (1) Hartigan	Limitation^a^	Inconsistency^d^	No Indirectness	Imprecision^d^	⨁◯◯◯ Very Low
Modified Cincinnati Knee Rating System	23 (1) Shaarani	Limitations^b^	Inconsistency^d^	No Indirectness	Imprecision^d^	⨁◯◯◯ Very Low
Return to Sport	23 (1) Shaarani	Limitations^b^	Inconsistency^d^	No Indirectness	Imprecision^d^	⨁◯◯◯ Very Low

^a^Lack of allocation concealment, lack of blinding and personnel, incomplete accounting of patients and outcome events

^b^Lack of blinding of participants and personnel, incomplete accounting of patients and outcome events

^c^Heterogeneity was considered large

^d^Only single trial available and < 400 participants so downgraded for inconsistency and imprecision

^e^Wide degree of variety in interventions and outcome measures

^f^Small sample sizes

### Pre-operative protocols

Pre-operative protocols differed across all three RCTs. Table 3 provides further detail regarding exercise programme content. The number of sessions varied from 10 to 24 and were completed over varying time frames. Hartigan, Axe and Snyder-Mackler [31] did not set participants a fixed number of sessions to complete per week only that ten sessions were to be completed, taking the perturbation group an average of 3.1- weeks to complete and the strength group 3.7-weeks. The two remaining studies specified the number of sessions to be completed each week; Shaarani et al. [27] four sessions a week for 6-weeks and Kim, Hwang and Park [28] three sessions a week for 4-weeks. The exercise interventions were predominantly completed with supervision. Shaarani et al. [27] specified two gym and two home sessions a week, Kim, Hwang and Park [28] had all sessions supervised in a sports medicine clinic and Hartigan, Axe and Snyder-Mackler [31] did not state whether sessions were supervised or completed at home. However, the perturbation group required a therapist to be involved in the intervention and it is therefore implied this group were supervised [31].

### Outcome measures

#### Muscle strength

All RCTs included quadriceps strength as an outcome measure but utilised different methods of assessment. Two RCTs reported a strength ‘index’ or ‘deficit’ as a percentage of the injured limbs force output compared to the uninjured limb; Hartigan, Axe and Snyder-Mackler [31] measured the highest quadriceps volitional isometric contraction reporting a ‘quadriceps strength index’ and Kim, Hwang and Park [28] measured power at angular speeds 60^o^/s and 180^o^/s reporting a ‘knee extensor strength deficit’. Shaarani et al. [27] measured quadriceps peak torque at an angular speed of 90^o^/s. As the measurements across studies were not comparable, the authors were not contacted for the raw data as data pooling would not have been possible.

Hartigan, Axe and Snyder-Mackler [31] found that quadriceps strength indexes improved in both groups from pre-intervention *(Pert: 87.2%; Str: 75.8%)* to six-month post-surgery *(Pert: 97.1%; Str: 94.4%)*. Although between group differences were not reported in their results.

Kim, Hwang and Park [28] reported that knee extensor strength deficits were significantly different between groups at both angular velocities 60°/s (*p* = 0.018) and 180°/s (*p* = 0.033) at follow-up and that the intervention group showed significantly greater improvements in post-operative strength than patients in the control. The authors did not provide point measures for the between group differences in knee extensor strength deficits from pre- to post-operation. The effect size was calculated (SMD) to be ‘small’ for PreHab at both angular velocities of 60°/s, 0.41 (95% CI − 0.85 to 0.01), and 180°/s, 0.23 (95% CI − 0.67 to 0.21). No minimal clinically significant difference (MCID) has been established for this outcome.

Shaarani et al. [27] found no statistical significance between the PreHab group and the control for quadriceps peak torque at any time point. This study also assessed hamstring peak torque [27] and again found no significant difference between groups for hamstring peak torque measured pre- and post-operatively.

#### Function

Two RCTs [27, 28] assessed single leg hop distance but reported results as a best of three score [27], or a limb symmetry index (LSI) (injured limb distance / uninjured limb distance) [28]. Both authors were contacted for the raw data, Shaarani et al. [27] provided this however, Kim, Hwang and Park [28] did not respond; data could therefore not be pooled for a meta-analysis. Both studies found that, at 12-weeks post-operation, single leg hop distance/LSI scores were significantly higher in the group who received PreHab compared to the control group. Both Kim, Hwang and Park [28] and Shaarani et al. [27] did not provide point measures for between group differences in single leg hop distance/LSI from pre- to post-operation.

The effect size (SMD) was ‘small’ for the single leg hop scores for PreHab in both studies; Kim, Hwang and Park [28], 0.48 (95% CI 0.48 to 0.03), and Shaarani et al. [27], 0.12 (95% CI − 0.77 to 0.99). No MCID has been reported for the single leg hop distance.

Gait was assessed by Hartigan, Axe and Snyder-Mackler [31] reporting knee excursion at the mid-stance of gait (obtained by calculating peak knee extension minus peak knee flexion). At six months post-surgery, the perturbation group showed no significant difference in knee excursion between limbs (*Mean: 3.58; 95% CI: 8.3 to − 1.4; p = 0.14)* whereas the strength group continued to show significant differences between limbs *(Mean 7.08; 95% CI: 11.6 to 2.5; p = 0.007).* No between group differences were reported.

#### Patient reported outcome measures

The Tegner score was reported to have been taken by Shaarani et al. [27] although no detail regarding the time points at which this was assessed, or scores obtained were reported in the published study or supplementary material. When contacted, it was confirmed that the Tegner-Lysholm Knee Score was assessed at all three time points (baseline, before ACLR and 12-weeks postoperatively). There were no statistically significant differences between the PreHab and control groups scores at any time point.

The Modified Cincinnati Knee Rating System was reported in one study [27]. The intervention group showed a statistically significant improvement from baseline (62.6) to the preoperative time point (76.5) (*p* = 0.004) to 12-weeks postoperatively (85.3) (*p* = 0.001). The mean score at 12-weeks postoperatively was also significantly higher (p = 0.004) for the PreHab group (85.3) compared with the controls (77.6). No between group differences were analysed.

#### Return to sport

Shaarani et al. [27] measured return to sport time in weeks following surgery. Although it was reported that the intervention group returned to sport sooner after surgery (mean time [SD], 34.18 weeks [4.14]) than the control group (42.5 weeks [4.14]), this difference was not statistically significant (*p* = 0.055). Shaarani et al. [27] also reported to have used the Tegner scale, but did not provide any results for this.

## Discussion

### Summary of Main findings

This systematic review demonstrates there is only limited, very low quality evidence to support the use of PreHab to improve knee extensor strength deficits, single leg hop distance/LSI, limb symmetry during gait and subjective knee scores for the Modified Cincinnati Knee Rating System after ACLR (3- and 6-months post-operatively). A clear limitation of this body of evidence is the small study sample sizes which are dominated by males (*n* = 116/122, 95%). Currently, no evidence exists to support the use of PreHab to improve return to preinjury levels of physical activity, function or psychological readiness post-surgery.

### Wider evidence base

Two RCTs in this review were also included in the 2017 Alshewaier, Yeowell and Fatoye [6] systematic review; Hartigan, Axe and Snyder-Mackler [31] and Shaarani et al. [27]. Alshewaier, Yeowell and Fatoye [6] assessed methodological quality using the Physiotherapy Evidence Base (PEDro) scale whereas the present review used the Cochrane RoB-2 tool. Both the PEDro and Cochrane Risk of Bias tools evaluate the risk of bias in RCTs and have six common items (random allocation, concealed allocation, blinding of participants, personnel and assessors, and incomplete outcome data), though it has been acknowledged that the tools cannot be used interchangeably and agreement between overall scores is poor [32]. The remaining studies included in the Alshewaier, Yeowell and Fatoye [6] review were excluded from this review due to study design (not RCT), data collection time-points (not all studies assessed participants post-operatively) and study population (not all participants underwent ACLR).

There are three remaining cohort studies in the literature that were excluded from both reviews. These studies report positive results for the effect of PreHab on post-operative objective and subjective outcomes, reporting improvements in International Knee Documentation Committee (IKDC) [33, 34], Knee Injury and Osteoarthritis Outcome Score (KOOS) [33, 34] and reduction in limb asymmetries [35] with PreHab. However, generalisability of results is limited as the study designs introduces a high risk of bias with key concerns including the risk of confounding, selection and information bias [36].

### Clinical and research implications

The evidence supporting the use of PreHab remains limited. In the included RCTs, no emphasis was placed on the importance of the psychological status of individuals prior to or following surgery and how PreHab may effect this; despite the evidence base identifying psychological barriers as the most commonly cited reasons for failing to return to physical activity after ACLR [12, 37, 38].

The results from one RCT [27] demonstrated that PreHab improved patient reported symptoms (Modified Cincinnati Knee Rating System) at 12-weeks post-operation. It has been suggested that increased subjective knee scores are associated with increased psychological readiness for return to activity [3, 14, 39]. Thus, it could be hypothesised that PreHab also improves psychological readiness, however further high-quality research needs to explore this more explicitly using validated outcome measures, such as the ACL-Return to Sport after Injury (ACL-RSI) scale [40].

The use of psychological factors to predict post-operative outcomes following ACLR and return to preinjury activity levels has frequently been cited in the literature [18, 41–44]. A case-control study of recreational and competitive level athletes established a link between pre- and early post-operative ACL-RSI scores and the likelihood of returning to preinjury activity, with higher scores favouring a return [3]. The generalisability of these results, however, is relatively limited due to study design, population and setting (private orthopaedic clinic). Further evidence has proposed a link between poor subjective knee scores within 1-year post ACLR and long-term impairments in health-related quality of life, emphasising further the importance of improving patients psychological response to surgery [45]. Aiming to improve psychological readiness and tackle patients fear of reinjury has been shown to support a return to preinjury levels of physical activity [42] and presents another unexplored function of PreHab.

Returning to preinjury levels of physical activity is a common goal for both patients and clinicians and often establishes the overall success of ACLR surgery [46]. Only one RCT assessed return to sport outcomes, reporting that the group who followed a preoperative programme returned to sport quicker than those in the control group (intervention 42.5 weeks [SD: 10.46], control 34.18 weeks [SD: 4.14]), although the difference did not reach statistical significance (*p* = 0.055).

Recent literature has emphasised the importance of evaluating post-operative progression against objective and time based criterion; a return to sport decision based on time alone is considered insufficient [9, 47]. A recent survey of Australian orthopaedic surgeons and physiotherapists found that when asked about return to sport time, a large proportion of both professions (77% surgeons, 78% physio) do not permit a return earlier than 9-months after ACLR [48, 49]. Returning to sport sooner than 9-months has also been suggested to increase the risk of reinjury [47] with some arguing the return should be no earlier than 12-months [50]. Shaarani et al. [27], did not define ‘return to sport’, and it is therefore unclear what level of activity participants returned to. The lead author was contacted for clarity regarding this outcome and provided their wider study material, but this detail was not included. The results should be translated with caution as no other detail regarding the success of participants in passing RTS objective and subjective criteria was provided.

There is a lack of consensus for clinicians on how best to deliver PreHab, exposing the potential for unnecessary time and cost being spent on this stage of rehabilitation. It is unknown how clinicians can optimally prepare patients both physically and mentally for surgery and return to physical activity.

### Study limitations

This review included a small number of RCTs of which all scored an overall high risk of bias with overall very low quality of evidence. It is likely that future studies would significantly alter our conclusion. Only one reviewer completed the searches in the electronic databases and of the unpublished literature, therefore eligible studies may have been missed. However, a systematic approach was taken to screen full text articles and reference list searching was completed to maximise the recognition of eligible studies. Only one reviewer extracted the data, although this was verified by a second reviewer. Meta-analysis was unable to be performed due to heterogeneity. Therefore, results are interpreted with caution.

## Conclusion

There is currently limited, very low quality evidence to support the use of PreHab for ACLR. The three included RCTs offer unconvincing results on post-operative outcomes of muscular strength, function and patient reported symptoms. Future research could look to provide consensus on the approach to PreHab and evaluate holistic interventions that consider the physical and psychological state of individuals and how this may affect post-operative biopsychosocial outcomes.

## Supplementary information

**Additional file 1: Supplementary File 1.** – PRISMA Statement. Completed PRISMA checklist 2009.

**Additional file 2: Supplementary File 2.** – Search Strategy. Database search strategy.

**Additional file 3: Supplementary File 3.** – GRADE Judgements. Additional GRADE judgement information.

**Additional file 4: Supplementary File 4.** – Excluded Studies. Full text articles excluded due to study design.

## Data Availability

All data generated or analysed during this study are included in this published article [and its supplementary information files].

## Abbreviations

ACL: Anterior cruciate ligament
ACLR: Anterior cruciate ligament reconstruction
ACL-RSI: ACL-Return to Sport after Injury
CSA: Cross sectional area
GRADE: Grading of recommendations assessment, development and evaluation
IKDC: International Knee Documentation Committee
KOOS: Knee Injury and Osteoarthritis Outcome Score
LSI: Limb symmetry index
MCID: Minimal clinically significant difference
MRI: Magnetic resonance imaging
MVIC: Maximum volitional isometric contraction
NPEG: No preoperative exercise group
PEG: Preoperative exercise group
PERT: Perturbation group
RCTs: Randomised controlled trials
ROB-2: Version 2 risk-of-bias
RTS: Return to sport
SD: Standard deviation
SMD: Standardised mean difference
STR: Strengthening group
