# Supplementary material for: The effectiveness of preoperative rehabilitation programmes on postoperative outcomes following anterior cruciate ligament (ACL) reconstruction: a systematic review
Source: BMC Musculoskelet Disord. 2020 Oct 3;21:647. doi: 10.1186/s12891-020-03676-6 (PMC7533034; doi:10.1186/s12891-020-03676-6)
Supplement: Supplementary file 2 — Additional file 2: Supplementary File 2. – Search Strategy. Database search strategy. [file 12891_2020_3676_MOESM2_ESM.docx]

## Additional File 2 – Search Strategy (EBSCOhost)

| 1 | Anterior cruciate ligament |
| --- | --- |
| 2 | ACL |
| 3 | Anterior cruciate ligament injury |
| 4 | Anterior cruciate ligament reconstruction |
| 5 | ACLR |
| 6 | Anterior cruciate ligament deficient |
| 7 | ACLD |
| 8 | OR / 1-7 |
| 9 | Pre-operative physiotherapy |
| 10 | Pre-operative protocol |
| 11 | Prehabilitation |
| 12 | Pre-operative rehabilitation |
| 13 | Pre-operative exercise |
| 14 | Pre-operative treatment |
| 15 | Psychological techniques |
| 16 | Health knowledge |
| 17 | Recovery expectation |
| 18 | Attitude to health |
| 19 | Locus of control |
| 20 | Anxiety |
| 21 | Depression |
| 22 | Self-efficacy |
| 23 | Kinesiophobia |
| 24 | Confidence |
| 25 | Motivation |
| 26 | Fear |
| 27 | Coping behaviour |
| 28 | Sport psychology |
| 29 | OR / 9-28 |
| 30 | Return to sport |
| 31 | Return to activity |
| 32 | Return to performance |
| 33 | Return to play |
| 34 | Postoperative outcomes |
| 35 | Athletic participation |
| 36 | Physical activity participation |
| 37 | Return to exercise |
| 38 | OR / 30 – 37 |
| 39 | 9 AND 29 AND 38  (limited to English and RCT design) |
