# Supplementary material for: The effectiveness of preoperative rehabilitation programmes on postoperative outcomes following anterior cruciate ligament (ACL) reconstruction: a systematic review
Source: BMC Musculoskelet Disord. 2020 Oct 3;21:647. doi: 10.1186/s12891-020-03676-6 (PMC7533034; doi:10.1186/s12891-020-03676-6)
Supplement: Supplementary file 3 — Additional file 3: Supplementary File 3. – GRADE Judgements. Additional GRADE judgement information. [file 12891_2020_3676_MOESM3_ESM.docx]

## Additional File 3 – GRADE Judgements

All studies were examined against the following criteria as per the GRADE handbook.

1. **Study limitations**

The following forms of bias were considered:

- 1. Selection bias – random sequence generation, allocation concealments, group similarities at baseline
  2. Performance bias – blinding of participants and/or healthcare providers
  3. Attrition bias – dropouts and intention-to-treat analysis
  4. Detection bias – blinding of the outcome assessors and timing of outcome assessments
  5. Reporting bias – selective reporting

Outcomes were downgraded by one level if any of the above levels of bias were detected.

1. **Inconsistency**

Outcomes were downgraded by one level if the heterogeneity was considered large. As the results were not pooled statistically, the following criteria was used:

- 1. Wide variance of point estimates across studies
  2. Minimal or no overlap of confidence intervals
  3. Statistical criteria, including tests of heterogeneity which tests the null hypothesis that all studies have the same underlying magnitude of effect, have a low p-value (p<0.05), indicating to reject the null hypothesis

1. **Indirectness**

The quality of evidence was assessed against the four sources of indirectness:

- 1. Differences in population
  2. Differences in interventions (applicability)
  3. Differences in outcome measures (surrogate outcomes)
  4. Indirect comparisons

Outcomes were downgraded by one level if any of the above were detected.

1. **Imprecision**

In general, results are considered imprecise when studies include relatively few patients and few events and therefore have a wide confidence interval around the estimate of effect. The following criteria was used:

Dichotomous outcomes:

- 1. If the optimal information size (OIS) criterion is not met (unless the sample size is very large e.g. > 2000)
  2. If the OIS criterion is met and the 95% confidence interval exclude no effect
  3. If the OIS criterion is met and the 95% confidence interval overlaps no effect

Continuous outcomes:

1. If the level of the 95% confidence interval includes no effect and the upper or lower limit crosses an effect size of 0.05 and 0.2.

The quality of evidence was downgraded by one for all outcomes as sample sizes were small across all studies (<400) and had few events (<300).

1. **Publication bias**

This domain was not assessed as funnel plot asymmetry should only be used when there are ten or more studies included.
