# Supplementary material for: The effectiveness of preoperative rehabilitation programmes on postoperative outcomes following anterior cruciate ligament (ACL) reconstruction: a systematic review
Source: BMC Musculoskelet Disord. 2020 Oct 3;21:647. doi: 10.1186/s12891-020-03676-6 (PMC7533034; doi:10.1186/s12891-020-03676-6)
Supplement: Supplementary file 4 — Additional file 4: Supplementary File 4. – Excluded Studies. Full text articles excluded due to study design. [file 12891_2020_3676_MOESM4_ESM.docx]

## Additional File 4 – Excluded Studies

[1] Arnold T, Shelbourne KD. A perioperative rehabilitation program for anterior cruciate ligament surgery. Phys Sportsmed 2000;28:31–44. https://doi.org/10.3810/psm.2000.01.621.

[2] Brand E, Nyland J. Patient outcomes following anterior cruciate ligament reconstruction: The influence of psychological factors. Orthopedics 2009;32:335. https://doi.org/10.3928/01477447-20090502-01.

[3] Failla MJ, Logerstedt DS, Grindem H, Axe MJ, Risberg MA, Engebretsen L, et al. Does Extended Preoperative Rehabilitation Influence Outcomes 2 Years after ACL Reconstruction? A Comparative Effectiveness Study between the MOON and Delaware-Oslo ACL Cohorts: Am J Sports Med 2016;44:2608–14. https://doi.org/10.1177/0363546516652594.

[4] Grindem H, Granan LP, Risberg MA, Engebretsen L, Snyder-Mackler L, Eitzen I. How does a combined preoperative and postoperative rehabilitation programme influence the outcome of ACL reconstruction 2 years after surgery? A comparison between patients in the Delaware-Oslo ACL Cohort and the Norwegian National Knee Ligament Registry. Br J Sports Med 2015;49:385–9. https://doi.org/10.1136/bjsports-2014-093891.

[5] Logerstedt D, Lynch A, Axe MJ, Snyder-Mackler L. Symmetry restoration and functional recovery before and after anterior cruciate ligament reconstruction. Knee Surgery, Sport Traumatol Arthrosc 2013;21:859–68. https://doi.org/10.1007/s00167-012-1929-2.

[6] Rodriguez RM, Marroquin A, Cosby N. Reducing fear of reinjury and pain perception in athletes with first-time anterior cruciate ligament reconstructions by implementing imagery training. J Sport Rehabil 2019;28:385–9. https://doi.org/10.1123/jsr.2017-0056.
